# Supplementary material for: Identifying Predictors for Minimum Dietary Diversity and Minimum Meal Frequency in Children Aged 6–23 Months in Uganda
Source: Nutrients. 2022 Dec 7;14(24):5208. doi: 10.3390/nu14245208 (PMC9786234; doi:10.3390/nu14245208)
Supplement: Supplementary file 1 [file nutrients-14-05208-s001.zip › nutrients-2001679-supplementary-1.pdf]

<u>Supplementary Material</u>

File S1

Construction of the female empowerment score

**Variable name**	**Aggregate codes used based on UDHS responses categories**
How woman's income is used	Code 1 when: the decision is taken by the woman or jointly with partner; otherwise, code 0.
How man's income is used	Code 1 when: the decision is taken by the woman or jointly with partner; otherwise, code 0.
Large household purchases	Code 1 when: the decision is taken by the woman or jointly with partner; otherwise, code 0.
Visiting family and friends	Code 1 when: the decision is taken by the woman or jointly with partner; otherwise, code 0.
Regarding own health care	Code 1 when: the decision is taken by the woman or jointly with partner; otherwise, code 0.
Woman salary similar/higher than man salary	Code 1 when: woman earns more than man, she is sole earner; otherwise, code 0.
Woman owns a land	Code 1 when: the woman or jointly with partner or alone & jointly owns a land; otherwise, code 0.
Woman owns a house	Code 1 when: the woman or jointly with partner or alone & jointly owns a house; otherwise, code 0.
Beating allowed if:<br>1. Goes out without telling him [male/husband];<br>2. Neglects the children;<br>3. Argues with him [male/husband];<br>4. Refuses to have sex with him [male/husband];<br>5. Burns the food	Code 1 when: the woman answered to all five questions 'No'; otherwise, code 0.
