# Supplementary material for: Identifying Predictors for Minimum Dietary Diversity and Minimum Meal Frequency in Children Aged 6–23 Months in Uganda
Source: Nutrients. 2022 Dec 7;14(24):5208. doi: 10.3390/nu14245208 (PMC9786234; doi:10.3390/nu14245208)
Supplement: Supplementary file 1 [file nutrients-14-05208-s001.zip › nutrients-2001679-supplementary-2.pdf]

File S2

Sample characteristics of children aged 6-23 months.

	**Sample N**	**Count (Percentage) or mean [SE]**
**Child characteristics**		
Birth order	5485	
Firstborn		1175 (21%)
Second to fourth		2490 (45%)
Fifth and more		1820 (33%)
Birth interval (months)	5393	
Firstborn		1175 (22%)
<24		1030 (19%)
>=24		3188 (59%)
Perceived birth weight/size	5485	
Smaller than average		1342 (25%)
Average		2710 (49%)
Larger than average		1336 (24%)
Unknown		97 (2%)
Received Vitamin A in the past 6 months	5148	3291 (64%)
Received iron pills, sprinkles or syrup in the last 7 days	5148	386 (8%)
**Maternal characteristics**		
Age (years)	5485	
15-24		2268 (41%)
25-34		2266 (41%)
35-49		951 (17%)
Delivered at health facility	5485	4198 (77%)
Type of delivery assistance	5485	
Health professional		4198 (77%)
Traditional birth attendant		1206 (22%)
Other		81 (2%)
Caesarean Delivery	5457	401 (7%)
Occupation	4670	
Not working		158 (3%)
Manual work		3453 (74%)
Non-manual work		1059 (23%)
Currently married or living with a partner	5485	4673 (89%)
Exposure to media: at least once a week	5485	
Reading newspaper		337 (6%)
Listening to radio		2994 (55%)
Watching TV		828 (15%)
Ethnicity	4707	
Bantu		2771 (59%)
Nilotics		804 (17%)
Nile Hermits		756 (16%)
Sudanics		248 (5%)
Other		128 (3%)
Religion	5485	
Anglican		1635 (30%)
Catholic		2282 (42%)
Muslim		748 (14%)
Other religion		820 (15%)
Slept last night under mosquito net	5485	3808 (70%)
**Paternal characteristics**		
Age (years)	4673	
15-24		599 (13%)

25-34		2100 (45%)
>=35		1974 (42%)
Highest educational level	4550	
No education		433 (10%)
Primary		2488 (55%)
Secondary or higher		1629 (36%)
Occupation	4661	
No occupation		149 (3%)
Manual work		3453 (74%)
Non-manual work		1059 (23%)
**Household characteristics**		
Female household head	5485	1269 (23%)
No. of household members	5485	6.1 [2.8]
No. of children under 5	5485	1.9 [0.9]
Types of cooking fuel	5485	
Electricity, LPG, natural gas, biogas		17 (0.3%)
Wood, straw/shrubs/grass, animal dung and other		5468 (99%)
Unimproved source of drinking water	5485	1166 (21%)
Source for water not in own dwelling or yard/plot	4263	4818 (96%)
Time to get to water source (min)	4707	
0 min		3 (0.1%)
1-59 mins		3097 (57%)
>= 60 mins		2385 (44%)
Unimproved toilet facility	4707	3673 (67%)
Shared toilet with other households	4240	2082 (44%)
