# Supplementary material for: Identifying Predictors for Minimum Dietary Diversity and Minimum Meal Frequency in Children Aged 6–23 Months in Uganda
Source: Nutrients. 2022 Dec 7;14(24):5208. doi: 10.3390/nu14245208 (PMC9786234; doi:10.3390/nu14245208)
Supplement: Supplementary file 1 [file nutrients-14-05208-s001.zip › nutrients-2001679-supplementary-4.pdf]

File S4

Sensitivity analysis: results from MICE imputation and complete case-analysis for all ages with a)models for MMF and b)for MDD indicators.

a) Models for MMF

	MICE imputation								Complete case-analysis							
**MINIMUM MEAL FREQUENCY**	**UNADJUSTED**				**ADJUSTED**				**UNADJUSTED**				**ADJUSTED**			
All ages	**OR (95% CI)**			**p value**	**OR (95% CI)**			**p value**	**OR (95% CI)**			**p value**	**OR (95% CI)**			**p value**
Vaccination status																
Not vaccinated	1				1				1				1			
Partially vaccinated	2.91 (2.28-3.72)	2.28	3.72	<0.001	2.45 (1.89-3.17)	1.89	3.17	<0.001	0.89 (0.55-1.45)	0.55	1.45	0.65	0.87 (0.53-1.42)	0.53	1.42	0.57
Fully vaccinated	4.04 (3.15-5.19)	3.15	5.19	<0.001	3.26 (2.48-4.28)	2.48	4.28	<0.001	1.02 (0.63-1.66)	0.63	1.66	0.94	0.95 (0.58-1.57)	0.58	1.57	0.85
Female empowerment																
Very low female empowerment	1				1				1				1			
Low female empowerment	1.08 (0.94-1.25)	0.94	1.25	0.3	1.08 (0.93-1.25)	0.93	1.25	0.29	1.07 (0.88-1.30)	0.88	1.30	0.50	1.06 (0.87-1.29)	0.87	1.29	0.57
Medium female empowerment	1.03 (0.87-1.21)	0.87	1.21	0.75	1.07 (0.91-1.27)	0.91	1.27	0.41	0.97 (0.79-1.20)	0.79	1.20	0.81	1.02 (0.82-1.27)	0.82	1.27	0.84
High female empowerment	0.84 (0.63-1.13)	0.63	1.13	0.25	0.90 (0.67-1.22)	0.67	1.22	0.51	0.72 (0.51-1.01	0.51	1.01	0.06	0.78 (0.55-1.11)	0.55	1.11	0.17
Wealth index																
First wealth percentile	1				1				1				1			
Second wealth percentile	1.34 (1.13-1.59)	1.13	1.59	<0.001	1.31 (1.10-1.56)	1.10	1.56	0.003	1.40 (1.14-1.72)	1.14	1.72	0.001	1.31 (1.06-1.62)	1.06	1.62	0.01
Middle wealth percentile	1.30 (1.09-1.55)	1.09	1.55	0.004	1.25 (1.04-1.51)	1.04	1.51	0.01	1.43 (1.15-1.77)	1.15	1.77	0.001	1.33 (1.07-1.66)	1.07	1.66	0.01
Fourth wealth percentile	1.45 (1.20-1.75)	1.20	1.75	<0.001	1.43 (1.17-1.76)	1.17	1.76	<0.001	1.74 (1.37-2.22)	1.37	2.22	<0.001	1.62 (1.26-2.10)	1.26	2.10	<0.001
Highest wealth percentile	1.43 (1.19-1.72)	1.19	1.72	<0.001	1.51 (1.18-1.92)	1.18	1.92	<0.001	2.08 (1.53-2.84)	1.53	2.84	<0.001	2.13 (1.49-3.03)	1.49	3.03	<0.001
Health status																
Not sick	1				1				1				1			
Sick	1.47 (1.30-1.66)	1.30	1.66	<0.001	1.25 (1.10-1.43)	1.10	1.43	<0.001	1.09 (0.93-1.29)	0.93	1.29	0.28	1.09 (0.93-1.29)	0.93	1.29	0.29

b) Models for MDD

				MICE imputation									Complete case-analysis				
**MINIMUM DIETARY DIVERSITY**	**UNADJUSTED**				**ADJUSTED**				**UNADJUSTED**				**ADJUSTED**			
All ages	**OR (95% CI)**			**p value**	**OR (95% CI)**			**p value**	**OR (95% CI)**			**p value**	**OR (95% CI)**			**p value**
Vaccination status																
Not vaccinated	1				1				1				1			
Partially vaccinated	4.28 (2.95-6.19)	2.95	6.19	<0.001	4.14 (2.82-6.06)	2.82	6.06	<0.001	1.59 (0.95-2.66)	0.95	2.66	0.08	1.51 (0.89-2.56)	0.89	2.56	0.13
Fully vaccinated	3.83 (2.63-5.56)	2.63	5.56	<0.001	3.97 (2.68-5.88)	2.68	5.88	<0.001	1.22 (0.73-2.06)	0.73	2.06	0.45	1.26 (0.74-2.15)	0.74	2.15	0.39
Female empowerment																
Very low female empowerment	1				1				1				1			
Low female empowerment	0.98 (0.85-1.14)	0.85	1.14	0.8	1.00 (0.86-1.17)	0.86	1.17	0.95	1.01 (0.83-1.23)	0.83	1.23	0.89	1.04 (0.85-1.26)	0.85	1.26	0.72
Medium female empowerment	0.97 (0.82-1.15)	0.82	1.15	0.75	1.12 (0.94-1.33)	0.94	1.33	0.22	1.02 (0.83-1.26)	0.83	1.26	0.83	1.15 (0.92-1.43)	0.92	1.43	0.22
High female empowerment	0.73 (0.53-1.02)	0.53	1.02	0.06	0.92 (0.66-1.28)	0.66	1.28	0.61	0.83 (0.57-1.19)	0.57	1.19	0.31	1.01 (0.69-1.47)	0.69	1.47	0.95
Wealth index																
First wealth percentile	1				1				1				1			
Second wealth percentile	1.43 (1.18-1.73)	1.18	1.73	<0.001	1.34 (1.11-1.63)	1.11	1.63	0.03	1.39 (1.11-1.73)	1.11	1.73	0.004	1.27 (1.01-1.59)	1.01	1.59	0.04
Middle wealth percentile	1.54 (1.27-1.87)	1.27	1.87	<0.001	1.44 (1.17-1.76)	1.17	1.76	<0.001	1.63 (1.30-2.05)	1.30	2.05	<0.001	1.49 (1.18-1.87)	1.18	1.87	<0.001
Fourth wealth percentile	2.03 (1.66-2.47)	1.66	2.47	<0.001	1.80 (1.45-2.22)	1.45	2.22	<0.001	2.25 (1.77-2.85)	1.77	2.85	<0.001	1.96 (1.53-2.53)	1.53	2.53	<0.001
Highest wealth percentile	2.51 (2.06-3.04)	2.06	3.04	<0.001	2.02 (1.57-2.59)	1.57	2.59	<0.001	3.00 (2.26-3.98)	2.26	3.98	<0.001	2.53 (1.83-3.51)	1.83	3.51	<0.001
Health status																
Not sick	1				1				1				1			
Sick	1.34 (1.18-1.54)	1.18	1.54	<0.001	1.25 (1.08-1.44)	1.08	1.44	0.002	1.05 (0.89-1.24)	0.89	1.24	0.55	1.09 (0.92-1.29)	0.92	1.29	0.33
