# Supplementary figures and images for: Identifying Predictors for Minimum Dietary Diversity and Minimum Meal Frequency in Children Aged 6–23 Months in Uganda
Source: Nutrients. 2022 Dec 7;14(24):5208. doi: 10.3390/nu14245208 (PMC9786234; doi:10.3390/nu14245208)

**File S3**

Percentage of frequency of foods eaten by children in the previous 24 hours.

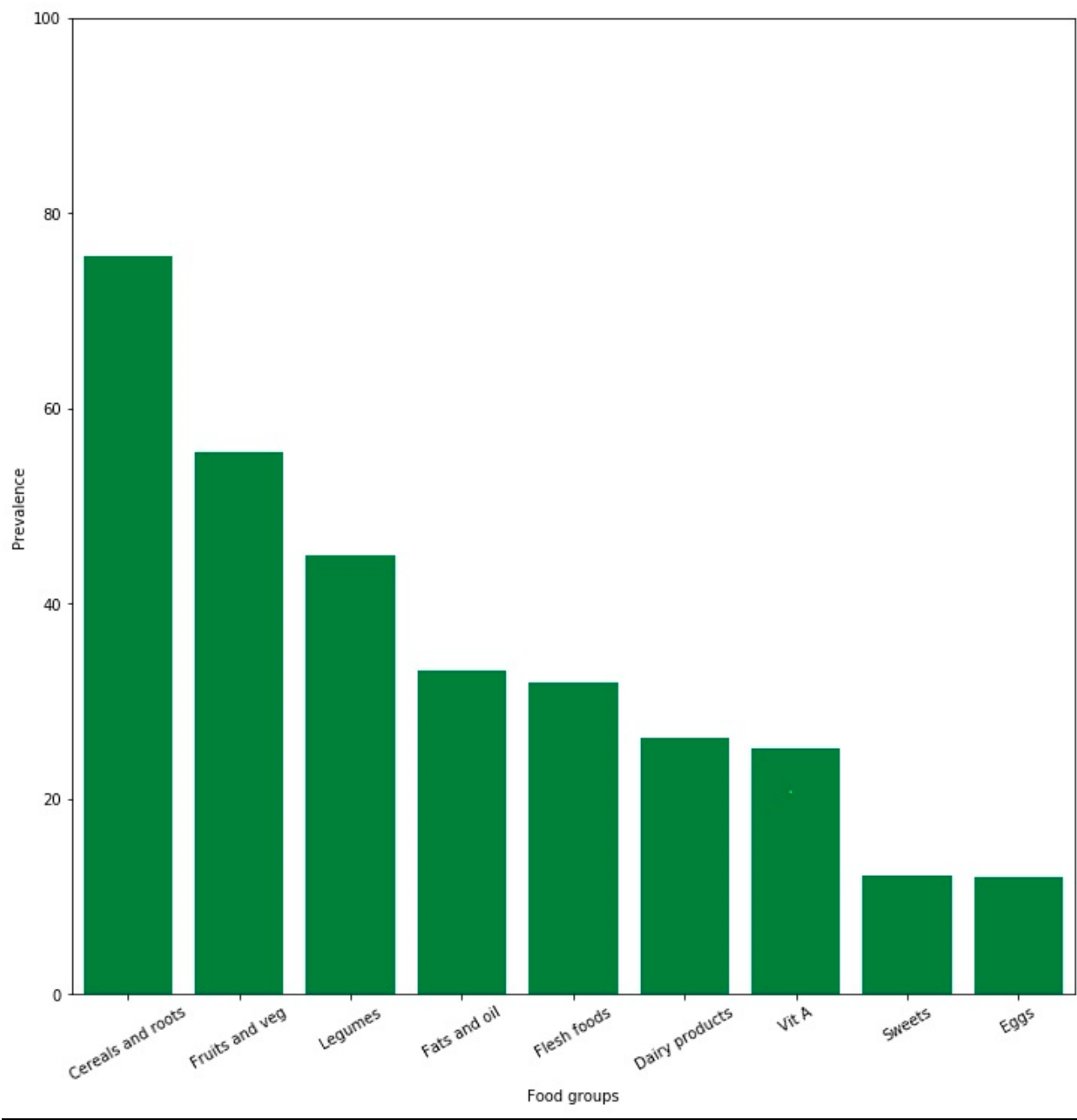

Supplement: Supplementary file 1 [file nutrients-14-05208-s001.zip › nutrients-2001679-supplementary-3.pdf]
